# Gait characteristics in people with central nervous system disorders under dual-task experimental paradigms: a scoping review

**DOI:** 10.3389/fnagi.2026.1807030

**Published:** 2026-10-07

**Authors:** Haizhe Jin, Jiahao Zhang, Xinyi Meng, Jianing Li, Su Meng, Quanwei Fu

**Affiliations:** 1 Department of Industrial Engineering, School of Business Administration, Northeastern University, Shenyang, Liaoning, China; 2 Department of Management Science and Engineering, School of Business Administration, Northeastern University, Shenyang, Liaoning, China; 3 Department of Industrial Engineering and Management, School of Business Administration, Northeastern University, Shenyang, Liaoning, China; 4 Department of Neurology, The First Hospital of China Medical University, Shenyang, Liaoning, China; 5 Guangdong Longcheng Hospital, Guangzhou, Guangdong, China; 6 CITIC Huizhou Hospital, Huizhou, Guangdong, China

**Keywords:** central nervous system diseases, cognitive–motor interference, dual-task cost, dual-task paradigm, gait analysis

## Abstract

**Background:**

Dual-task (DT) paradigms are widely used for gait assessment in patients with central nervous system diseases. However, previous studies have primarily focused on specific diseases and a limited range of DT paradigms, making it difficult to obtain an overall understanding of the application patterns of different paradigms and their associated gait changes. This scoping review aimed to identify and classify the DT paradigms used in DT gait studies of Alzheimer’s disease (AD), Parkinson’s disease (PD), and stroke; describe the application and distribution of these paradigms across studies of different diseases; further examine changes in gait characteristics under different DT paradigms.

**Methods:**

The Web of Science Core Collection, PubMed, and the Cochrane Library were systematically searched for relevant studies published between January 1, 2005, and April 30, 2026. DT paradigms were classified, and their distribution was mapped by disease category. Dual-task cost (DTC) for gait speed, stride length, and cadence was also summarized descriptively.

**Results:**

A total of 84 studies were included, and 26 DT paradigms were identified, including 21 cognitive tasks, four motor tasks, and one combined cognitive–motor task. Serial subtraction by 3s and 7s were more frequently used in studies of PD, whereas the counting task was more frequently used in studies of AD. The verbal fluency task was relatively evenly distributed across studies of the three diseases, whereas the object-carrying task was used primarily in studies of PD and stroke. Among the paradigms included in the descriptive synthesis, mean gait speed DTC was numerically highest under the verbal fluency task in patients with AD, under serial subtraction by 7s in patients with PD and healthy controls, and under the counting task in patients with stroke. Available data on stride length and cadence were relatively limited.

**Conclusion:**

The paradigms used in DT gait studies of AD, PD, and stroke were primarily concentrated in a small number of commonly used paradigms, and their distribution was uneven across diseases. The descriptive DTC findings suggest that patterns of gait change may differ according to disease and DT paradigm.

## 1 Introduction

Central nervous system (CNS) diseases refer to a class of diseases in which impairment of the structure and function of the central nervous system leads to cognitive, motor, and other functional deficits, and common types include Alzheimer’s disease (AD), Parkinson’s disease (PD), and stroke (Kim and Fraser, 2022). Patients with these diseases often present with symptoms such as memory decline, gait abnormalities, bradykinesia, and dystonia in daily life, which severely affect their activities of daily living and quality of life (Ogawa et al., 2018; Burtscher et al., 2024; Khalili et al., 2024; Tenchov et al., 2024), and increase caregiving and economic burdens (Low et al., 1999; Adal et al., 2024). Current studies indicate that the clinical symptoms of CNS diseases may change as these diseases progress, and assessing their clinical progression is therefore important for safeguarding patients’ quality of life (Oh et al., 2021; Kim et al., 2024).

Gait impairment is a common clinical manifestation in patients with CNS diseases (Mirelman et al., 2019; Andrade-Guerrero et al., 2024). Patients may exhibit reduced gait speed, shortened stride length, decreased cadence, and increased gait variability (Longhurst et al., 2023; Russo et al., 2025). Gait analysis, as an objective and quantitative assessment method, can assess gait changes under cognitive tasks, motor tasks, and combined cognitive–motor tasks, and has the advantages of being non-invasive and easy to implement (Blumen et al., 2023; Russo et al., 2025; Nedović et al., 2024; Nedović et al., 2025). A large number of studies have shown that gait characteristics are closely associated with disease characteristics, and the gait characteristics of patients with CNS diseases are often used in studies of clinical subgroup classification and disease progression assessment (Montero-Odasso and Hachinski, 2014; Wang et al., 2023; Burtscher et al., 2024). For example, Kim et al. conducted a 2-year longitudinal study of patients with PD and found that the dual-task (DT) effect on arm swing asymmetry during DT walking was associated with subsequent cognitive decline, suggesting the potential value of specific DT gait parameters for monitoring disease progression (Kim et al., 2024). Russo et al. developed classification models using spatiotemporal gait parameters collected from patients with PD under single-task (ST), motor DT, and cognitive DT conditions. The results showed that these gait characteristics could distinguish patients with PD who had mild cognitive impairment (MCI) from those who did not, and that the cognitive DT condition yielded a greater number of key classification features (Russo et al., 2023).

In gait research involving patients with CNS diseases, the DT paradigm has been widely used (Raffegeau et al., 2019; Tsang et al., 2022; Wang et al., 2023). In this context, DT involves walking while simultaneously performing a cognitive task (e.g., the verbal fluency task [VFT], serial subtraction) or a motor task (e.g., an object-carrying task, an obstacle negotiation task). For example, participants may perform serial subtraction by 7s (SS7) while walking, a condition referred to as SS7-DT. Compared with ST walking, the additional task in DT may compete with walking for processing resources, potentially reducing the resources available for walking and impairing gait performance. In other words, under DT, gait abnormalities may become more apparent, which may help detect gait changes that are less apparent during ST walking (Andrade-Guerrero et al., 2024).

This study selected AD, PD, and stroke as representative types of CNS diseases. These three diseases have a relatively substantial evidence base in DT gait research and may differ in their physiological characteristics, underlying mechanisms, and cortical function. Specifically, the physiological characteristics of AD may include progressive cognitive decline and structural degeneration of the brain. In a study of patients with amnestic MCI and AD, Wang et al. found that fallers had lower gray matter volumes in the cerebellum, fusiform gyrus, and parts of the temporal lobe (Wang et al., 2026). The potential mechanism underlying gait impairment may involve cognition and walking competing for limited attentional resources. As cognitive impairment worsens, the ability to recruit additional cortical resources to maintain gait may become constrained. Regarding cortical function, Teo et al. found that individuals with subjective memory decline exhibited increased activation in the left prefrontal cortex during DT walking, whereas prefrontal activation decreased in the dementia group (Teo et al., 2021). Impaired gait automaticity may be a physiological characteristic of PD. Maidan et al. found that patients with PD exhibited greater prefrontal activation even during usual walking and that patterns of cortical recruitment differed across DT conditions. This may reflect an increased reliance on cognitive control and prefrontal resources to compensate for impaired gait automaticity (Maidan et al., 2016). Stroke may be physiologically characterized by focal lesions and interhemispheric network reorganization. Liu et al. found that cognitive and motor DT conditions further recruited the contralesional supplementary motor area and bilateral premotor areas in patients after stroke, although the specific recruitment patterns differed across DT conditions. This may reflect the recruitment of bilateral and contralesional motor-related networks to meet the increased motor-control and cognitive-processing demands imposed by different DT conditions (Liu et al., 2018). These findings provide a theoretical basis for considering disease-related differences in neural control when interpreting gait performance under DT conditions. Cortical activation and neural network recruitment during DT walking may also help elucidate how brain resources are allocated during walking.

Gait characteristics may vary across populations with CNS diseases and DT conditions (Tsang et al., 2022; Wang et al., 2023). For example, Yang et al. conducted buttoning-task and tray-carrying DT assessments in patients with stroke, and the results showed that, in the buttoning-task DT condition, only stride length was significantly lower in patients with stroke than in healthy controls (HC), whereas during the tray-carrying DT condition, gait speed, stride length, and the temporal symmetry index were all significantly lower in patients with stroke than in HC (Yang et al., 2007). Subotic et al. conducted a counting task (CNT) during walking (CNT-DT) in three groups of patients, including patients with PD without cognitive impairment, patients with PD with MCI, and a combined group comprising patients with PD dementia and patients with dementia with Lewy bodies, and the results showed that gait speed differed significantly among the three patient groups under both ST and DT conditions (Subotic et al., 2024). These studies indicate that in addition to differences observed within each condition, there are also differences across neurological conditions.

Studies with similar research objectives may nevertheless use different DT paradigms, gait parameters, and statistical evaluation metrics. For example, with the same objective of AD identification, Li et al. studied patients with AD under ST and SS7-DT conditions and used a dimensionality-reduction method to group 20 original gait parameters into several gait domains. The results showed that stride asymmetry under ST, as well as rhythm and variability under SS7-DT, could distinguish patients with AD from HC; however, identification accuracy did not differ significantly between the two conditions (Li et al., 2023). Oh et al. conducted a study similar to that of Li et al. but used ST and VFT-DT conditions and assessed gait speed, stride length, and double-support time. The results showed that, compared with the ST baseline model, significant improvements in model fit for AD identification were observed only when stride length and double-support time under VFT-DT were included in the model (Oh, 2021). Similarly, with the same objective of distinguishing between groups with different cognitive statuses, Callisaya et al. assessed gait and cognitive task performance in participants with dementia, MCI, and HC under an alternating alphabet recitation DT condition. They found that cognitive-task performance differed among the groups, whereas ST gait speed, DT gait speed, and the percentage of DT interference in gait speed did not differ significantly (Callisaya et al., 2025). Montero-Odasso et al. assessed gait in participants with dementia, MCI, and HC under VFT-DT conditions and reported that DT gait speed could be used to classify participants into groups (Montero-Odasso et al., 2017). Taken together, these studies show that even when research objectives are similar, experimental conditions, gait parameters, and statistical evaluation metrics may differ across studies. Consequently, the reported findings may capture different dimensions of gait and reflect differences in the statistical evaluation metrics used. Therefore, comparisons of findings across studies should take into account the study population, DT paradigm, gait parameters, and statistical evaluation metrics.

Previous studies addressing cognitive impairment in patient populations and the cognitive demands of DT have examined DT gait performance in relation to task type, cognitive load, and the complexity of the primary walking task. Galletly and Brauer compared arithmetic, verbal, and motor tasks in patients with PD and found that gait changes varied across DT conditions (Galletly and Brauer, 2005). Plummer-D’Amato et al. distinguished among working memory, visuospatial judgment, and spontaneous speech tasks in patients with stroke and concurrently assessed gait performance and cognitive task performance (Plummer-D’Amato et al., 2008). Muir et al. further compared three cognitive DT conditions in individuals with normal cognition and patients with MCI or AD to examine how cognitive status and task type were associated with gait performance (Muir et al., 2012). In addition, Oh et al. established conditions with no, low, and high cognitive loads and used repeated assessments to examine changes in multiple gait parameters across different load levels (Oh et al., 2021). More recently, Conklin et al. considered both the complexity of the primary walking task and the type of secondary task in their experimental design (Conklin et al., 2026). However, these studies varied in their DT paradigms, gait parameters, and research objectives, and the available evidence remains scattered across studies involving different diseases and task types. Currently, there is a lack of systematic mapping of DT paradigms, their task characteristics, and their application and distribution across studies of AD, PD, and stroke, together with a cross-disease synthesis of gait changes reported under selected commonly used paradigms. This gap makes it difficult for researchers to obtain an overall understanding of the disease populations and task contexts covered by the available evidence and the associated gait changes, thereby complicating the interpretation and comparison of individual study findings within the broader evidence base.

In summary, this study used a scoping review approach to identify and classify the DT paradigms used in DT gait studies of AD, PD, and stroke; describe the application and distribution of these paradigms across studies of different diseases; further examine changes in gait characteristics under different DT paradigms; and provide a reference for understanding the potential relationships among disease characteristics, DT paradigm characteristics, and gait performance. Scoping reviews are appropriate for research fields characterized by broad research questions and substantial heterogeneity in the existing evidence. They enable the systematic mapping of the scope and distribution of different types of evidence and the synthesis and classification of key concepts and research methods (Munn et al., 2018). This synthesis is intended to provide context for understanding possible relationships among disease characteristics, DT paradigm characteristics, and gait performance, and to inform the design and interpretation of future DT gait studies. The findings of this study are primarily applicable to DT gait studies involving patients with AD, PD, and stroke.

## 2 Methods

### 2.1 Databases, research scope, and search strategy

To ensure the comprehensiveness and systematicity of the literature search, this study selected Web of Science Core Collection, PubMed, and the Cochrane Library for literature retrieval. Based on the purpose and scope control of this review, the review focused on patients with AD, PD, or stroke.

The construction of search terms was based on the population, intervention, comparator, and outcome framework: (1) Population: patients with AD, PD, or stroke; (2) Intervention: DT; (3) Comparator: ST; and (4) Outcomes: gait parameters. The search used a combination of Medical Subject Headings (MeSH) and free-text terms to maximize coverage of relevant literature. The specific search terms and search strategies are shown in Table 1.

**Table 1:** Search strategy.

Database	Search in	Search terms
PubMed	Title/Abstract	("Parkinson Disease"[MeSH] OR "Parkinson" OR "Parkinson's disease " OR "Stroke"[MeSH] OR "cerebrovascular accident" OR "Alzheimer Disease"[MeSH] OR "Alzheimer" OR "Alzheimer's disease") AND ("Gait"[MeSH] OR "walking" OR "locomotion" OR "Ambulation" OR "Kinematics") AND ("multitasking behavior"[MeSH] OR "Dual Task" OR "Dual-Task" OR "cognitive-motor")
Cochrane Library	Title/Abstract/Keyword	
Web of Science Core Collections	Topic	

MeSH, Medical Subject Headings. Controlled vocabulary (MeSH) was used when available (e.g., “Parkinson Disease”[MeSH], “Stroke”[MeSH], “Alzheimer Disease”[MeSH], “Gait”[MeSH]), combined with free-text terms.

### 2.2 Inclusion and exclusion criteria

This study developed the following inclusion and exclusion criteria: (1) only peer-reviewed original research was included, including randomized controlled trials, cohort studies, cross-sectional studies, and case–control studies; (2) the language was limited to English; (3) the search period was set from January 1, 2005, to April 30, 2026; (4) participants had a confirmed diagnosis of AD, PD, or stroke. Considering that MCI has frequently been examined as part of the cognitive continuum in AD-related DT gait research, data from MCI groups were also extracted when these groups were included in AD-related studies and their data could be separated from those of other diagnostic groups; (5) studies had to report gait parameters during walking tasks and provide comparative data under ST and DT conditions; (6) reviews, meta-analyses, conference abstracts, editorials, letters, research posters, and other non-journal publications were excluded; (7) studies involving other CNS diseases (e.g., multiple sclerosis) or mixed diagnoses were excluded when data for eligible participant groups could not be extracted separately; and (8) studies that did not report gait parameter data or from which ST–DT comparative results could not be extracted were excluded.

### 2.3 Study selection

First, the records obtained from the initial search were imported into EndNote X9, and duplicate records were automatically removed. Then, two researchers independently conducted study selection: (1) initial screening: titles and abstracts were read, and clearly irrelevant studies (e.g., animal experiments, and articles on engineering/device development) were excluded according to the inclusion and exclusion criteria; (2) full-text screening: the full texts of reports that passed the initial screening were read, and studies that did not meet the criteria were further excluded according to the inclusion and exclusion criteria. If disagreements occurred during screening, they were resolved through discussion; if consensus could not be reached, a third researcher adjudicated. Finally, a total of 84 studies that met the criteria were included. The whole process and results of study selection (including the reasons and numbers for exclusion at each stage) were presented with reference to the Preferred Reporting Items for Systematic Reviews and Meta-Analyses extension for Scoping Reviews (PRISMA-ScR) flow diagram (Tricco et al., 2018), as shown in Figure 1.

**Figure 1:**
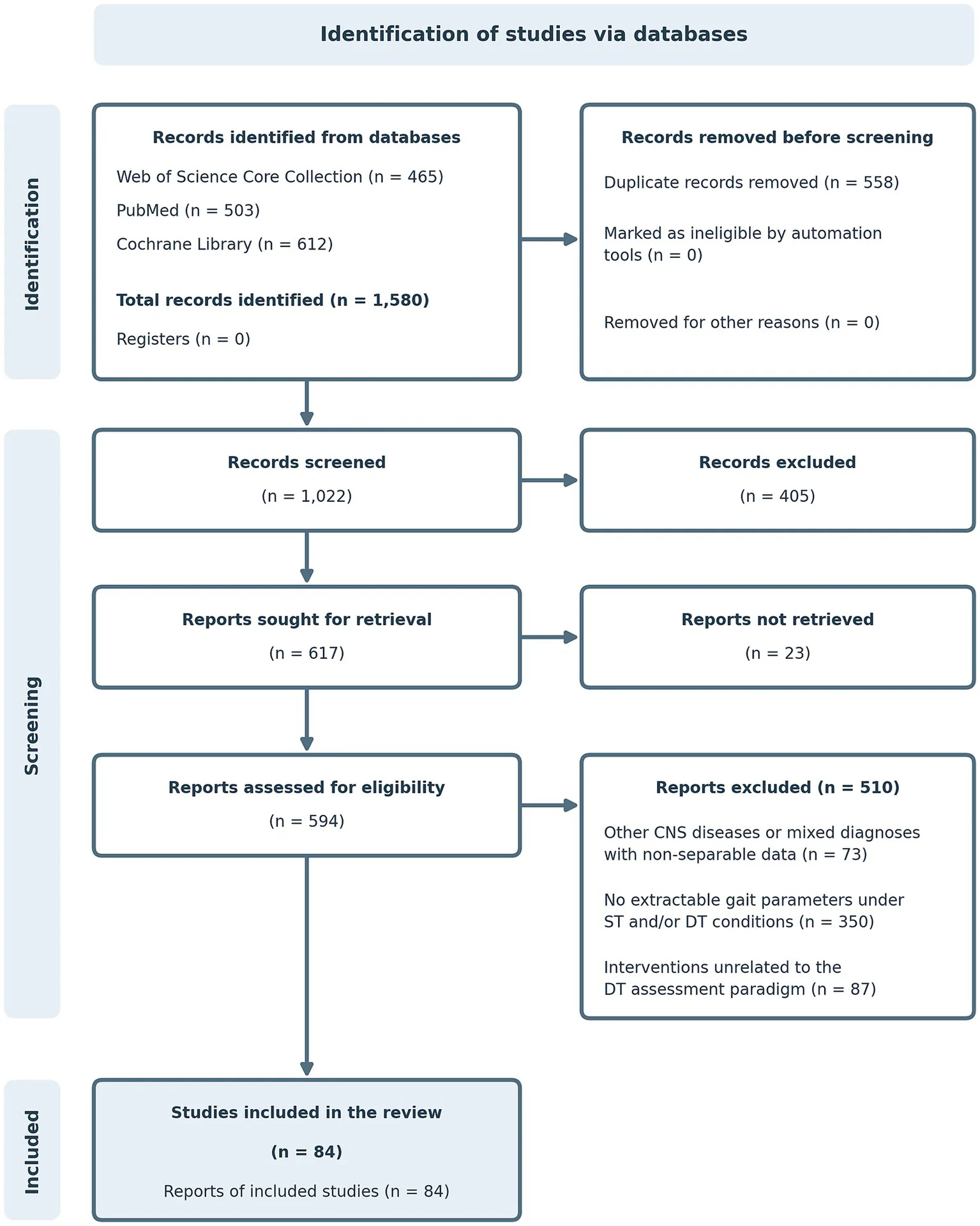
PRISMA-ScR flow diagram of study selection. The diagram summarizes identification, screening, eligibility, and inclusion steps for this review. PRISMA-ScR = preferred reporting items for systematic reviews and meta-analyses extension for scoping reviews. A total of 1,580 records were identified, and 84 studies were included in the review.

As shown in Figure 1, the study selection process fully presents the entire process from literature retrieval to final study inclusion. First, a total of 1,580 records were identified through the initial search. After 558 duplicate records were removed, 1,022 records remained. Second, according to the inclusion and exclusion criteria described above, 405 records that did not meet the criteria were excluded after title and abstract screening, leaving 617 reports for full-text retrieval. Third, during the full-text retrieval stage, we attempted to obtain the full texts of these reports through university library resources, PubMed, Google Scholar, ResearchGate, and other platforms. However, the full texts of 23 reports could not be retrieved due to access restrictions, full-text unavailability, or related reasons, leaving 594 reports. Finally, the remaining 594 reports were assessed through full-text screening strictly according to the inclusion and exclusion criteria. A total of 510 reports were excluded, and 84 studies were ultimately included in the review.

### 2.4 Data extraction and data analysis

For data extraction, this study extracted basic information from the 84 included studies, including author, publication year, country, disease type, sample size, sex, age, and cognitive level, as well as study population, DT paradigm, and gait parameters.

During gait parameter selection, this study selected gait speed, stride length, and cadence, which were relatively commonly reported gait parameters. In addition, gait parameters such as swing speed, stance time, and gait cycle were also occasionally reported in relevant studies. Given their less frequent reporting, these additional parameters were outside the scope of the descriptive synthesis.

During DT paradigm selection, this study summarized and classified the extracted DT paradigms, and counted the use of different DT paradigms in previous studies. The subsequent descriptive synthesis focused on four commonly used non-interactive cognitive DT paradigms—serial subtraction by 3s (SS3), SS7, CNT, and VFT—to examine gait parameter changes across paradigms with broadly similar task characteristics.

For gait parameter analysis, this study used dual-task cost (DTC) to quantify changes in gait parameters of patients with CNS diseases and HC under different DT conditions. Gait DTC refers to the percentage change in a given gait parameter, including gait speed, stride length, or cadence, under DT relative to the ST condition. Based on the calculation method reported by Muir et al. (2012), gait DTC was calculated using the following equation:


$$\mathrm{DTC}(\%)=\frac{\left(\text{Performanc}e_{\mathrm{ST}}-\text{Performanc}e_{\mathrm{DT}}\right)}{\text{Performanc}e_{\mathrm{ST}}}\times 100\%$$

Where $\text{Performanc}e_{\mathrm{DT}}$ denotes task performance under the DT condition, and $\text{Performanc}e_{\mathrm{ST}}$ denotes task performance under the ST condition. A positive DTC indicates that performance under DT was lower than that under ST, a DTC of 0 indicates no change, and a negative DTC indicates that performance under DT was higher than that under ST.

DTC data were synthesized descriptively across participant groups and DT paradigms. To ensure the stability and interpretability of the comparative results as much as possible, studies used for comparing gait parameters were further selected from the 84 included studies based on the following criteria: (1) the study reported at least one of the commonly used DT paradigms selected for the main analysis; (2) the study reported at least one of the three selected gait parameters and provided the corresponding DTC or gait parameter data under ST and DT conditions that could be used to calculate DTC; and (3) the reported data could be classified into at least one of the following participant groups: AD, PD, stroke, or HC. Subsequently, to systematically characterize the distribution of DTC across different research contexts, the extracted data were cross-classified in Microsoft Excel 2021 according to four participant groups, four DT paradigms, and three gait parameters, yielding a total of 48 possible “participant group–DT paradigm–gait parameter” combinations. For combinations with extractable data, the means, standard deviations, and ranges were calculated. Table 2 presents descriptive DTC summaries for patients with CNS diseases and HC under the selected DT conditions. These analyses were intended to provide an exploratory descriptive summary of DTC across different research contexts.

**Table 2:** Descriptive statistics for the dual-task costs of spatiotemporal gait parameters across dual-task paradigms and participant groups.

Gait DTC	DT paradigm	AD				PD				Stroke				HC			
		Mean	SD	Range	k/n	Mean	SD	Range	k/n	Mean	SD	Range	k/n	Mean	SD	Range	k/n
Gait speed DTC	SS3	22.00%	–	–	1/1	12.64%	0.0489	3%−21%	13/16	18.35%	0.0480	13%−25%	5/5	8.45%	0.0362	4%−14%	5/6
	SS7	26.68%	0.0488	22%−31%	4/4	18.24%	0.0481	9%−29%	9/17	–	–	–	0/0	13.98%	0.0414	9%−21%	7/7
	CNT	16.56%	0.0436	12%−25%	5/5	9.00%	0.0141	8%−10%	2/5	27.42%	0.0684	23%−32%	2/3	5.92%	0.0595	0%−15%	6/6
	VFT	27.42%	0.0718	20%−37%	6/7	18.00%	0.0847	0%−21%	6/8	14.89%	0.0565	7%−35.5%	5/5	10.14%	0.0598	6%−19%	7/7
Stride length DTC	SS3	9.00%	–	–	1/1	9.90%	0.0524	4%−17%	10/11	11.61%	0.0032	11%−12%	3/3	5.22%	0.0247	0%−7%	4/5
	SS7	10.50%	0.0354	8%−13%	3/3	11.45%	0.0329	7%−17%	5/11	–	–	–	0/0	6.50%	0.0342	3%−11%	3/3
	CNT	11.50%	0.0354	9%−14%	2/2	–	–	–	0/0	–	–	–	0/0	1.50%	0.0071	1%−2%	2/2
	VFT	15.33%	0.0635	8%−19%	3/3	12.00%	0.0600	5%−16%	3/3	11.05%	0.0583	5%−21%	3/3	6.01%	0.0335	3%−10%	3/3
Cadence DTC	SS3	16.00%	0.1273	7%−25%	2/2	4.36%	0.0364	0%−7%	7/9	10.01%	0.0369	7%−14%	3/3	3.30%	0.0324	−1%−7%	3/4
	SS7	24.00%	–	–	1/1	4.46%	0.0248	1%−8%	4/10	–	–	–	0/0	12.00%	–	–	1/1
	CNT	13.00%	–	–	1/1	–	–	–	0/0	–	–	–	0/0	3.50%	0.0354	1%−6%	4/4
	VFT	32.00%	–	–	1/1	7.00%	0.0240	5%−10%	3/3	13.00%	–	–	1/1	8.55%	0.0586	3%−15%	3/3

DT, dual task; DTC, dual-task cost; AD, Alzheimer’s disease; PD, Parkinson’s disease; HC, healthy controls; *k*, number of independent studies; *n*, number of DTC estimates; SD, standard deviation; SS3, serial subtraction by 3s; SS7, serial subtraction by 7s; CNT, counting task; VFT, verbal fluency task. “–” indicates not reported. The mean values above were calculated from the DTC estimates for the corresponding participant group, DT paradigm, and gait parameter across the 52 studies.

Meta-analysis generally requires relevant studies to be sufficiently comparable in terms of populations, interventions or exposures, comparison conditions, and outcome measures, thereby allowing a summary effect and its uncertainty to be estimated through the weighted pooling of effect sizes. An exploratory descriptive synthesis was selected instead of meta-analysis in view of the following evidence characteristics and the objectives of this review. First, the evidence was markedly unevenly distributed across different combinations of diseases, DT paradigms, and gait parameters. Some specific combinations were supported by only one study, whereas others had no relevant studies, resulting in limited evidence for some comparisons. Second, the included studies differed substantially in disease stage, subgroup definitions, cognitive status, DT implementation, task-prioritization instructions, gait-assessment devices, and the definitions, calculations, and reporting methods used for gait parameters, thereby limiting comparability across studies. Finally, some studies reported multiple participant subgroups, DT conditions, or gait outcomes, meaning that the resulting data were not statistically independent. Furthermore, the primary objective of this review was to identify and classify the DT paradigms used in gait studies of AD, PD, and stroke and describe their application and distribution across these diseases. Therefore, considering both the objectives of this study and the characteristics of the available evidence, a scoping review was more appropriate.

### 2.5 Methodological quality and risk of bias

All 84 included studies underwent methodological appraisal. First, the studies were classified according to their respective study designs: 72 analytical cross-sectional studies, four cohort studies, five diagnostic test accuracy studies, two quasi-experimental studies, and one randomized controlled trial. The corresponding JBI critical appraisal tools were then applied to each study design (Barker et al., 2023, 2024, 2025, 2026; Campbell et al., 2015). Two reviewers independently conducted the appraisals. Any disagreements were initially resolved through discussion between the two reviewers. If consensus could not be reached, a third reviewer was consulted to make the final decision.

## 3 Results

### 3.1 Characteristics of included studies

Table 3 summarizes the characteristics of the 84 included studies, including author, publication year, country, disease type, clinical information, sample size, sex distribution, age, and cognitive status.

**Table 3:** Characteristics of included studies.

Number	Author (year)	Country	Disease type	Clinical information	Sample size	Male/female	Age	Cognitive level
1	Zhou et al. (2024)	Taiwan, China	PD	–	14	8/6	62.29 ± 5.37	MMSE: 29.00 ± 1.57
2	Zhang et al. (2023)	China	PD	–	33	14/19	64.27 ± 8.53	MoCA: 24.15 ± 3.84
3	Kazemi et al. (2024)	Iran	PD	–	36	17/19	63.63 ± 8.91	MoCA: 26.94 ± 2.64
4	Kim et al. (2024)	USA	PD	Cognitively stable; disease duration 78.1 ± 38.1 months	30	17/13	61.5 ± 8.8	MoCA: 27.5 ± 2.1
			PD	Cognitive decline; disease duration 62.8 ± 26.8 months	18	9/9	67.6 ± 5.6	MoCA: 24.2 ± 2.6
5	Fernández-Lago et al. (2024)	Spain, Hungary	PD	–	44	33/11	63.1 ± 9.65	MMSE: 28.2 ± 1.72
6	Graham et al. (2023)	UK, USA	PD	FoG+	22	16/6	68.41 ± 7.87	MoCA: 27.05 ± 2.77
			PD	FoG−	21	11/10	69.33 ± 4.97	MoCA: 27.62 ± 2.22
			HC	–	17	9/8	69.24 ± 7.97	MoCA: 27.65 ± 1.69
7	Russo et al. (2023)	Italy	PD	–	40	–	Internal dataset: 61.32 ± 8.10; External dataset: 63.55 ± 11.19	–
8	Parati et al. (2022)	Italy	PD	–	25	21/4	69 ± 7	MMSE ≥ 24
9	Zhang et al. (2024)	China	PD	With anxiety	38	13/25	64	MMSE: 25
			PD	Without anxiety	106	67/39	67.5	MMSE: 27
10	Prell et al. (2022)	Germany	PD	–	40	23/17	70.65 ± 8.83	MoCA: 22.08 ± 3.94
11	Shkury et al. (2023)	Israel	PD	Idiopathic	33	22/11	67.69 ± 9.693	MoCA: 25.97 ± 2.279
12	Flugon et al. (2022)	Norway	AD	–	99	45/54	71.9 ± 9.3	MMSE: 24
13	Rice et al. (2022)	USA	Stroke	–	24	15/9	56 ± 12.9	MoCA: 23 ± 4.8
			HC	–	17	8/9	57 ± 9.6	
14	Muci et al. (2022)	Turkey, Northern Cyprus	Stroke	–	37	25/12	54.03 ± 13.50	MMSE: 26.49 ± 3.58
			HC	–	38	25/13	54.66 ± 9.87	MMSE: 28.21 ± 2.20
15	Ansai et al. (2017)	Brazil	MCI	–	40	6/34	75.8 ± 6.3	–
			AD	–	38	16/22	77.7 ± 6.2	–
16	Abdollahi et al. (2024)	USA	Stroke	–	21	11/10	66 ± 10	–
			HC	–	20	8/12	60 ± 8	–
17	Alcock et al. (2016)	UK	PD	–	76	50/26	67.5	MMSE: 28.6
			HC	–	81	39/42	69.7	MMSE: 29.2
18	Amatachaya et al. (2016)	Thailand	Stroke	Disease duration: 83.6 ± 70.4 months	51	23/28	64.2 ± 7.8	MMSE: 21.88 ± 5.33
			Stroke	Disease duration: 64.9 ± 52.4 months	54	27/27	60.1 ± 8.3	MMSE: 24.8 ± 3.74
19	Baetens et al. (2013)	Belgium	Stroke	Disease duration: 10.6 ± 6.7 weeks	32	22/10	64.8	–
20	Chatterjee et al. (2018)	USA	Stroke	Disease duration: 57.55 ± 50.58 months	9	5/4	60.55 ± 10.58	MMSE ≥ 21
		USA	Stroke	Disease duration: 30.16 ± 34.88 months	31	19/12	57.96 ± 9.49	MMSE ≥ 21
21	Oh (2021)	USA	AD	–	14	5/9	78.03 ± 12.06	DRS-2: 88.14 ± 7.07
			HC	–	15	5/10	72.71 ± 11.86	MoCA: 27.73 ± 1.29
22	Oh et al. (2021)	USA	AD	Disease duration: 5.11 years	14	5/9	78.03	DRS-2: 88.14
23	Denneman et al. (2018)	Netherlands	Stroke	Disease duration: 31.9 ± 19.7 days	78	49/29	59.1 ± 10.8	–
24	Dennis et al. (2009)	UK	Stroke	Disease duration: 25 months	21	13/8	61 ± 12	MMSE: 27.1
			HC	–	10	8/2	60 ± 6	–
25	Stegemöller et al. (2014)	USA	PD	Disease duration: 4.8 ± 4.5 years	35		66.2 ± 8.5	MMSE: 29.2 ± 1.0
26	Feld et al. (2018)	USA	Stroke	Disease duration: 8.9 months	28	17/11	58.2 ± 16.6	–
27	Galletly and Brauer (2005)	Australia	PD	Disease duration: 9.1 ± 4.5 years	16	9/7	65 ± 9.5	MMSE: 28 ± 3
			HC	–	16	9/7	65 ± 9.6	MMSE: 30 ± 1
28	Harrison et al. (2017)	USA	PD	Disease duration: 3.8 ± 4.2 years	23	13/10	69.5 ± 7.6	MMSE: 29
29	Hyndman et al. (2006)	UK	Stroke	Disease duration: 16 months	36	21/15	66.5 ± 11.8	–
			HC	–	24	14/10	62.3 ± 11.61	–
30	König et al. (2017)	France	AD	–	23	12/11	77 ± 9	MMSE: 17 ± 4.6
			MCI	–	24	8/16	75 ± 9	MMSE: 24.8 ± 3.2
			HC	–	20	5/15	73 ± 7	MMSE: 28.35 ± 1.5
31	Lee et al. (2015)	South Korea	Stroke	Disease duration: 5.22 ± 3 years	13	9/4	54.77 ± 13.08	MMSE: 27.00 ± 1.91
		South Korea	Stroke	Disease duration: 4.06 ± 3.08 years	15	9/6	53.53 ± 14.75	MMSE: 27.00 ± 1.96
		South Korea	Stroke	Disease duration: 5.52 ± 7.17 years	18	10/8	49.78 ± 11.15	MMSE: 28.61 ± 2.03
32	Lin et al. (2016)	China	AD	–	10	2/8	74.0 ± 8.6	MMSE: 17.7 ± 4.1
			HC	–	10	2/8	73.8 ± 6.1	MMSE: 29.4 ± 0.7
33	Lohnes and Earhart (2011)	USA	PD	Disease duration: 9.09 ± 5.39 years	11	4/7	70.27 ± 6.80	–
			OLDER HC	–	11	4/7	70.82 ± 10.44	–
			YOUNGER HC	–	11	4/7	24.09 ± 0.83	–
34	Lord et al. (2010)	UK, New Zealand	PD	Disease duration: 5.8 ± 5.5 years	29	19/10	71.3 ± 7.4	MMSE: 26.9 ± 2.8
35	Lord et al. (2006)	New Zealand	Stroke	Disease duration: 45.8 ± 34.2 months	27	20/7	61 ± 11.6	MMSE ≥ 24
36	Muir et al. (2012)	Canada	AD	–	23	9/14	77.5 ± 5.0	MMSE: 24.2 ± 2.3
			MCI	–	29	12/17	73.6 ± 6.2	MMSE: 27.5 ± 1.9
			HC	–	25	3/22	71.0 ± 5.0	MMSE: 29.5 ± 0.6
37	Plotnik et al. (2011)	Israel	PD	Disease duration: 9.4 ± 4.6 years	30	21/9	65.9 ± 7.2	MMSE: 28.5 ± 1.3
38	Plotnik et al. (2009)	Israel, USA	PD	–	21	15/6	71.9 ± 7.3	–
			HC	–	13	6/7	68.4 ± 4	–
39	Plummer-D’Amato et al. (2008)	USA	Stroke	Disease duration: 8.7 months	14	11/3	60.5 ± 15.3	MMSE: 26.7 ± 2.7
40	Rochester et al. (2014)	UK	PD	Disease duration: 4 months	121	81/40	67.0 ± 10.4	MMSE: 28.6 ± 1.3
			HC	–	184	78/106	69.4 ± 7.7	MMSE: 29.2 ± 1.0
41	Gillain et al. (2016)	Belgium	MCI	MCI+	9	4/5	74.44 ± 4.16	MMSE: 26.11 ± 1.45
			MCI	MCI-	4	2/2	70.00 ± 2.16	MMSE: 27.25 ± 1.70
42	Salazar et al. (2017)	USA	PD	Disease duration: 4.9 ± 4.2 years	19	11/8	66.3 ± 5.6	–
			HC	–	13	5/8	63.2 ± 4.5	–
43	Yogev-Seligmann et al. (2013)	Israel, USA	PD	Hoehn & Yahr stage 2-3	18	16/2	68.7 ± 4.7	MoCA: 25.6 ± 1.7
			YOUNGER HC	–	21	11/10	26.4 ± 2.1	–
			OLDER HC	–	15	6/9	75.4 ± 5.4	MoCA: 27 ± 1.7
44	Yogev-Seligmann et al. (2012)	Israel, USA	PD	Disease duration: 7.4 ± 4.6 years	20	12/8	70.3 ± 6.6	MoCA: 26.4 ± 2.8
			OLDER HC	–	20	10/10	70.9 ± 7.0	MoCA: 26.4 ± 1.9
45	Shimura et al. (2021)	Japan	AD	–	21	8/13	79.03 ± 6.89	MMSE: 19.62 ± 4.71
46	Sobol et al. (2016)	Denmark	AD	–	185	104/81	70.4 ± 7.3	MMSE: 24.5
47	Toda et al. (2019)	Japan	PD	–	64	37/27	67.9 ± 8.6	MMSE: 28.9 ± 1.4
			HC	–	20	11/9	71 ± 7.1	–
48	Wild et al. (2013)	Brazil	PD	Disease duration: 8.39 ± 2.85 years	18	8/10		–
			HC	–	18	8/10	69.44 ± 1.41	–
49	Yang et al. (2016)	Taiwan, China	Stroke	Disease duration: 105.9 ± 61.6 months	88	64/24	62.6 ± 7.8	–
50	Yang et al. (2007)	Taiwan, China	Stroke	Disease duration: 20.93 ± 11.15 months	15		61.27 ± 6.69	–
		Taiwan, China	Stroke	Disease duration: 20.00 ± 19.03 months	15		61.93 ± 13.48	–
		Taiwan, China	HC	–	15		62.73 ± 9.02	–
51	Yogev et al. (2005)	Israel	PD	–	30		70.9 ± 7.9	MMSE: 28.1 ± 1.6
			HC	–	28		69.8 ± 6.3	MMSE: 29.1 ± 1.1
52	Li et al. (2023)	China	AD	–	27	8/19	67.70 ± 8.12	MMSE: 15
			MCI	–	35	14/21	65 ± 9.04	MMSE: 26
			HC	–	40	18/22	60.58 ± 8.48	MMSE: 29
53	Lander et al. (2025)	USA	PD	Disease duration: 5.4 ± 4.7 years	19	6/13	66.2 ± 5.8	MoCA: 26 ± 3.3
54	Kitago et al. (2025)	Japan	Stroke	Disease duration: 76.0 ± 39.5 days	30	23/7	65.4 ± 13.5	MMSE: 27.7 ± 2.2
55	Ghislieri et al. (2025)	Italy	PD	–	27	19/8	57.4 ± 7.8	–
			HC	–	30	12/18	55.0 ± 8.8	–
56	Di Filippo et al. (2025)	Italy	PD	–	41		61.39 ± 8.17	–
57	Zhou et al. (2025)	Taiwan, China	PD	Hoehn-Yahr stages I–III	30	18/12	64.27 ± 7.39	MMSE ≥ 24
58	ALMohiza and Reddy (2026)	Saudi Arabia	PD	Hoehn-Yahr stages I–II	46	28/18	66.42 ± 7.81	MoCA: 25.38 ± 2.71
59	Caronni et al. (2025)	Italy	PD	Disease duration: 6 years; Hoehn-Yahr stages I–III	33	18/15	71	Frontal Assessment Battery: median16.5 (14.25–17)
60	Kim et al. (2026)	South Korea	PD	Disease duration: 4.4 ± 1.9 years; Hoehn-Yahr stages ≤II	30	9/21	70.17 ± 9.23	MMSE: 27.13 ± 2.74
			YOUNGER HC	–	30	12/18	25.13 ± 3.17	MMSE: 29.80 ± 0.41
			OLDER HC	–	30	15/15	73.47 ± 6.65	MMSE: 28.00 ± 1.02
61	Katagiri et al. (2025)	Japan	MCI	–	18	10/8	72.2 ± 4.9	MMSE: 27.3 ± 1.7
			HC	–	18	8/10	69.7 ± 5.9	MMSE: 28.0 ± 1.3
62	Conklin et al. (2026)	USA	PD	Hoehn-Yahr stages I–III	41	24/17	68.05 ± 2.39	MMSE: 27.98
			HC	–	11	6/5	66.73	MMSE: 28.73
63	Molinero-Martín et al. (2026)	Spain	PD	Disease duration: 6.57 ± 3.65 years; Hoehn-Yahr stages I–III	30	13/17	59.93 ± 10.97	MoCA: 24.40 ± 3.87
64	Mohammadi et al. (2025)	Iran	AD	–	24	9/15	74.0 ± 5.3	MMSE: 18.6 ± 4.5
			amnestic MCI	–	25	11/14	73.8 ± 5.5	MMSE: 25.5 ± 2.6
			HC	–	26	15/11	69.3 ± 4.4	MMSE: 28.5 ± 1.1
65	Liu et al. (2022)	Taiwan, China	PD	–	28	17/11	66.1 ± 8.0	MMSE: 28.5 ± 1.4
66	Pieruccini-Faria et al. (2023)	Canada	Stroke	–	123	87/36	69.0 ± 7.4	MoCA: 25.4 ± 2.9
67	Chatterjee et al. (2019)	USA	Stroke	Disease duration: 19.2 ± 10.4 months	33	22/11	59.6 ± 9.7	MMSE: 26.6 ± 3.1
68	Hermand et al. (2020)	France	Stroke	Acute; Disease duration: 54.5 ± 39.3 days	21	14/7	68.1 ± 9.4	–
			Stroke	Subacute; Disease duration: 68.1 ± 28.2 days	–	–	–	–
69	Hermand et al. (2019)	France	Stroke	Acute; Disease duration: 45.5 ± 34.5 days	11	6/5	71.4 ± 10.1	–
			Stroke	Acute; Disease duration: 45.5 ± 34.5 days	11	6/5	71.4 ± 10.1	–
70	Maidan et al. (2016)	Netherlands	PD	Disease duration: 9.1 ± 0.7 years	68	46/22	71.6 ± 0.9	MMSE: 28.2 ± 0.2
			HC	–	38	20/18	70.4 ± 0.9	MMSE: 28.8 ± 0.2
71	Liu et al. (2021)	China	MCI	–	20		62.41 ± 5.03	MMSE: 27.64 ± 1.76
72	Liu et al. (2018)	Taiwan, China	Stroke	Disease duration: 41.5 months	23	21/2	51.5	MMSE: 27.8 ± 2.2
73	Rosenberg-Katz et al. (2016)	Israel	PD	PIGD	30	18/12	64.95 ± 7.71	–
			PD	TD	30	23/7	64.60 ± 8.85	–
			PD	Uncertain patients	45	9/36	65.16 ± 8.43	–
			HC	–	28	16/12	64.91 ± 8.76	MMSE>24
74	Sartor et al. (2017)	Germany	PD	–	22	13/9	62	MMSE: 29
			PD	–	15	11/4	75	MMSE: 28
			HC	–	65	33/32	57	MMSE: 29
			HC	–	36	21/15	77	MMSE: 28
75	Stuart and Mancini (2020)	USA	PD	FoG−	12	9/3	68.67 ± 3.85	MoCA: 28.25 ± 3.62
			PD	FoG+	13	8/5	69.69 ± 4.21	MoCA: 26.08 ± 3.62
76	Subotic et al. (2024)	Canada	PD	–	19	18/1	72.7 ± 8.7	MoCA: 19.0 ± 4.4
			PD WITH MCI	–	18	15/3	70.1 ± 8.1	MoCA: 22.5 ± 4.2
			HC	–	20	5/15	68.7 ± 5.7	MoCA: 27.5 ± 1.8
77	Teo et al. (2021)	Singapore	Dementia	–	9	5/4	86.1 ± 7.3	MoCA: 20.3 ± 3.5
			SMC	–	23	10/13	73.5 ± 6.7	MoCA: 27.8 ± 1.9
			HC	–	26	14/12	71.1 ± 4.2	MoCA: 28.1 ± 1.6
78	Wang et al. (2022)	China	Cognitive impairment	–	16	4/12	69.68 ± 6.52	MMSE: 22.93 ± 2.13
			HC	–	38	11/27	67.92 ± 7.44	MMSE: 28.36 ± 1.58
79	Weng et al. (2023)	China	MCI	–	17	4/13	71	MMSE: 26
			HC	–	17	1/16	70	MMSE: 28
80	Lench et al. (2025)	USA	PD	FoG+	38	27/11	67.1 ± 7.1	MMSE: 28.5 ± 1.5
			PD	FoG−	17	14/3	68.6 ± 5.3	MMSE: 28.8 ± 1.3
81	Kvist et al. (2026)	Sweden	PD	Hoehn & Yahr stages I–IV	37	21/16	69 ± 7	–
			HC	–	44	25/19	69 ± 7	–
82	Grosboillot et al. (2025)	France	Stroke	Disease duration: 42.7 ± 21.8 days	33	20/13	72.9 ± 10.2	–
83	Binder et al. (2026)	Germany	Stroke	Disease duration: 24 ± 56 months	12	6/6	64.1 ± 7.4	–
			HC	–	19	5/14	71.3 ± 6.6	–
84	Wang et al. (2026)	China	AD	–	21	–	–	MMSE: 17.7 ± 0.9
			Amnestic MCI	–	24	–	–	MMSE: 23.9 ± 0.28

PD, Parkinson’s disease; AD, Alzheimer’s disease; MCI, mild cognitive impairment; SMC, subjective memory complaint; HC, healthy controls; MMSE, Mini-Mental State Examination; MoCA, Montreal Cognitive Assessment; DRS-2, Dementia Rating Scale-2; FoG+, freezing of gait positive; FoG−, freezing of gait negative; PIGD, postural instability and gait difficulty; TD, tremor dominant. “–” indicates not reported.

In terms of geographical distribution, the 84 included studies represented 26 countries or regions. The largest number of studies was from the United States (*n* = 21), followed by China, including Taiwan (*n* = 14). Seven studies were from Israel, six from the United Kingdom, five from Italy, and four each from Japan and France. Canada and Germany each contributed three studies. Belgium, Brazil, Iran, the Netherlands, New Zealand, South Korea, and Spain each contributed two studies. Australia, Denmark, Hungary, Northern Cyprus, Norway, Saudi Arabia, Singapore, Sweden, Thailand, and Turkey each contributed one study. For multinational or multiregional studies, each study was counted once for every country or region involved; therefore, the sum of studies across all countries or regions exceeded the total number of included studies.

In terms of study populations, PD-related studies were the most numerous, with 43 studies (51.2%). Stroke-related studies followed, with 23 studies (27.4%). AD-related studies accounted for 18 studies (21.4%).

In terms of clinical information, some studies stratified disease characteristics. Some PD studies additionally focused on freezing of gait, comorbid anxiety status, cognitive status, motor subtypes, and disease severity. Some stroke studies additionally focused on disease subtypes and disease stage.

In terms of sample size, 52 studies had sample sizes of fewer than 50 participants, 22 studies had sample sizes of 50–100 participants, and 10 studies had sample sizes of more than 100 participants.

In terms of sex distribution, the proportion of males was higher in PD- and stroke-related studies, with 20.0%–94.7% for PD and 45.1%–91.3% for stroke, while the proportion of females was higher in AD-related studies, with 37.5%–85.0%.

In terms of age characteristics, patients with AD were mainly older adults, and the age distribution was dispersed, with an overall range of 62.41–86.1 years. Patients with PD were mainly middle-aged and older adults, and the age distribution was concentrated, with an overall range of 57.4–75.0 years. Patients with stroke had the widest age span, covering young and middle-aged adults to older adults, and had the lowest mean age, with an overall range of 49.78–72.9 years.

In terms of cognitive status, the most commonly used cognitive assessment tool was the Mini-Mental State Examination (MMSE), followed by the Montreal Cognitive Assessment (MoCA), and 2 AD-related studies also used the Dementia Rating Scale-2. Cognitive function in patients with PD was generally well preserved, with an MMSE range of 24.0–29.2 and a MoCA range of 19.0–28.25, mainly showing MCI. Cognitive status in patients with stroke varied greatly, with an MMSE range of 21.0–29.2 and a MoCA range of 23.0–25.4, and was related to lesion location and disease duration. Cognitive status in patients with AD was relatively low, with an MMSE range of 15.0–27.64 and a MoCA range of 20.3–27.8, mainly showing moderate-to-severe cognitive impairment.

### 3.2 Methodological quality and risk of bias

The 84 included studies were appraised according to their respective study designs: 72 analytical cross-sectional studies, four cohort studies, five diagnostic test accuracy studies, two quasi-experimental studies, and one randomized controlled trial. The appraisal identified several methodological strengths. First, all 72 analytical cross-sectional studies clearly described the single-task and dual-task conditions, used standardized outcome measurement methods, and reported appropriate statistical analyses. Second, the four cohort studies used consistent outcome measurement methods and reported the duration of follow-up. Third, all five diagnostic test accuracy studies used accepted clinical or neuropsychological reference standards. Finally, the two quasi-experimental studies and the randomized controlled trial also used consistent outcome measurement methods.

Several methodological limitations were also identified. First, among the analytical cross-sectional studies, 51 of 72 explicitly reported their eligibility criteria, 41 clearly reported objective diagnostic criteria, and 48 described the study participants and settings in sufficient detail. In addition, 12 studies did not explicitly report strategies for addressing relevant confounding factors. Second, among the four cohort studies, only one clearly reported complete follow-up. Third, all five diagnostic studies evaluated the ability of gait parameters to distinguish between patients with an established diagnosis and healthy controls. This two-gate approach may introduce patient selection bias and overestimate diagnostic performance. Furthermore, the studies did not clearly report whether the gait parameters were interpreted without knowledge of the clinical diagnosis or whether the clinical reference classification was determined without knowledge of the gait findings. Fourth, although the randomized controlled trial reported random group allocation, it did not describe the specific method used to generate the random sequence or the procedures used to conceal allocation. Moreover, because participants and intervention providers were aware of group assignment, the study may have been susceptible to performance bias. The complete study-level appraisal is provided in Supplementary Table S1.

### 3.3 DT types

In this section, the DT paradigms used in the 84 included studies were summarized; the details are shown in Table 4.

**Table 4:** Dual-task paradigms involved in gait studies of central nervous system diseases.

DT	Task type	Explanation	Study No. (Table 3)
SS3	Non-interactive cognitive	Participants are required to start from a specified number (e.g., 100), subtract 3 sequentially, and continuously report the calculation results aloud.	1, 3, 4, 8, 19, 24, 25, 27, 37, 49, 54, 57, 60, 61, 63, 65, 70, 72, 73, 78, 79, 84
VFT	Non-interactive cognitive	Participants are required to continuously say corresponding words orally within a given theme (e.g., “animals”) (e.g., “monkey”).	12, 14, 19, 20, 21, 22, 27, 28, 33, 36, 43, 44, 45, 49, 55, 58, 66, 71, 84
SS7	Non-interactive cognitive	Participants are required to start from a specified number (e.g., 100), subtract 7 sequentially, and continuously report the calculation results aloud.	2, 7, 13, 36, 37, 38, 45, 48, 51, 52, 56, 67, 74, 77, 78, 80, 47, 59
CNT	Non-interactive cognitive	Participants are required to start from a specified number (e.g., 100), subtract 1 sequentially, and continuously report the results aloud.	9, 16, 18, 21, 22, 30, 36, 41, 46, 62, 64, 71, 76
TMT-B	Non-interactive cognitive	Participants are required to alternately say number and letter sequences (e.g., 3-C-4-D-5-E).	10, 42, 62
Spontaneous speech	Non-interactive cognitive	Participants are required to freely express thoughts, experiences, feelings, etc. orally, and maintain fluent and coherent speech.	39, 62
Alternate-letter task	Non-interactive cognitive	Participants are required to walk while orally producing letters according to an alternating-letter rule.	80
Backward month recitation	Non-interactive cognitive	Participants are required to take “January” as the starting point and orally report all month names in reverse order (e.g., January →December →November…→February).	46
Backward spelling task	Non-interactive cognitive	Participants are required to walk while spelling a given word backward.	53
Listening to music	Non-interactive cognitive	Participants are required to listen to prescribed or self-selected music accompaniment (e.g., “Row, Row, Row Your Boat”) and walk to the rhythm.	28
Singing	Non-interactive cognitive	Participants are required to sing a prescribed song with accompaniment or a cappella (e.g., “Row, Row, Row Your Boat”), maintain pitch accuracy and rhythmic continuity, and complete the whole song or a segment.	28
Digit recall task	Interactive cognitive	Participants are required to listen to randomly generated digit strings played from a loudspeaker and orally repeat them in the original order.	6, 17, 23, 32, 34, 40
Phoneme monitoring	Interactive cognitive	Participants are required to listen to a speech material; when the target phoneme is detected, timely feedback is given via button press, verbal response, etc.	5, 11, 48, 51, 83
n-back	Interactive cognitive	Participants are required to judge, in a series of stimuli (e.g., numbers, letters), whether the current stimulus is the same as the nth previous stimulus.	39, 68, 69, 82
Stroop task	Interactive cognitive	Participants are required to listen to auditory stimuli with semantic conflict (e.g., hearing the pronunciation of “red” but actually referring to blue), ignore the interference, and judge and answer accurately.	26, 81
Text comprehension	Interactive cognitive	Participants are required to read or listen to a material and then answer relevant questions, or complete tasks such as sentence cloze, logical judgment, etc.	48, 51
Visuospatial decision task	Interactive cognitive	Participants are required to listen to an audio clip describing a time every 5 s (e.g., “10 past 3”, “25 to 7”) and quickly and accurately judge whether the hour and minute hands on the clock face are on the same side or different sides at the given time.	24, 39
Auditory reaction-answering task	Interactive cognitive	Participants are required to listen to auditory stimuli such as questions and instructions and then verbally answer “yes” to represent “red” and “no” to represent “blue”.	31
AX-CPT	Interactive cognitive	Participants are required to listen to randomly presented letters; whenever the letter “I” appears immediately after the letter “A”, they press a button by hand.	75
Memory task	Interactive cognitive	Participants are required to complete a memory task for the experimental materials.	29
Odd-even judgment task	Interactive cognitive	Participants are required to judge odd or even after hearing a number and answer aloud (once every 10 s).	35
Phone dialing task	Combined cognitive–motor	Participants are required to answer the phone, and during the call they may maintain the call or end the call as needed.	15
Object carrying	Motor	Participants are required to perform object-carrying behaviors while walking, such as holding a tray with a glass cup with both hands or holding a glass cup filled with water; dropping the cup or spilling water is considered a failure.	1, 7, 14, 31, 34, 49, 50, 56, 57, 59, 61, 65, 72
Obstacle negotiation task	Motor	Participants are required to step over 6 evenly spaced obstacles (e.g., shoes) placed on the walking path, maintain the walking rhythm, and avoid touching the obstacles.	20, 35
Button pressing	Motor	Participants are required to press the electronic counter button as fast as possible with the dominant thumb.	27
Buttoning	Motor	Participants are required to button four shirt buttons with the dominant hand; failure is defined if the task is not completed.	50

DT, dual task; SS3, serial subtraction by 3s; SS7, serial subtraction by 7s; CNT, counting task; VFT, verbal fluency task; AX-CPT, AX Continuous Performance Test; TMT-B, Oral Trail Making Test Part B.

As shown in Table 4, previous gait studies on AD, PD, and stroke used a total of 26 DT paradigms. In these DT paradigms, the primary task was walking, and the concurrent tasks included 21 cognitive tasks such as SS3, SS7, and VFT, 4 motor tasks such as object carrying, buttoning, and obstacle negotiation, and the cognitive–motor combined task of phone dialing.

In the comparative analysis of the 21 cognitive tasks, we found that, according to whether participants interacted with external information during the experiment, these tasks could be divided into non-interactive cognitive tasks and interactive cognitive tasks. Non-interactive cognitive tasks mean that participants obtain the rules and content of the cognitive task before the experiment starts, and they do not need to obtain additional information during the experiment. For example, in SS7, an initial value and calculation rules are given at the beginning of the experiment, and participants report the calculation results by themselves during the experiment, without obtaining additional information. Interactive cognitive tasks mean that participants obtain the rules of the cognitive task before the experiment starts, and obtain the content of the cognitive task through interaction with external information during the experiment, that is, they respond in real time to external stimuli (e.g., visual or auditory stimuli). For example, in the n-back task, participants continuously receive auditory stimuli during walking and judge whether the current stimulus is the same as the stimulus presented n trials earlier. Similarly, there were also some differences among the four motor tasks. Some motor tasks are completed by body parts unrelated to walking, such as carrying a cup of water and button-pressing, while other motor tasks are completed by the lower limbs, such as obstacle negotiation.

As shown in Table 4, VFT, SS3, SS7, and CNT were commonly used non-interactive cognitive DT paradigms and provided extractable data for the subsequent descriptive synthesis. To reduce differences in task characteristics and resource demands between cognitive and motor tasks and improve comparability across paradigms, the subsequent exploratory descriptive synthesis of DTC was restricted to these four DT paradigms. Although the object-carrying task has been commonly used in previous studies, it was not included in this comparison because it is a motor DT and differs in its task characteristics from the cognitive DT paradigms described above.

### 3.4 Application and distribution of dual-task paradigms across central nervous system diseases

As shown in Figure 2, the 84 included studies involved a total of 26 DT paradigms.

**Figure 2:**
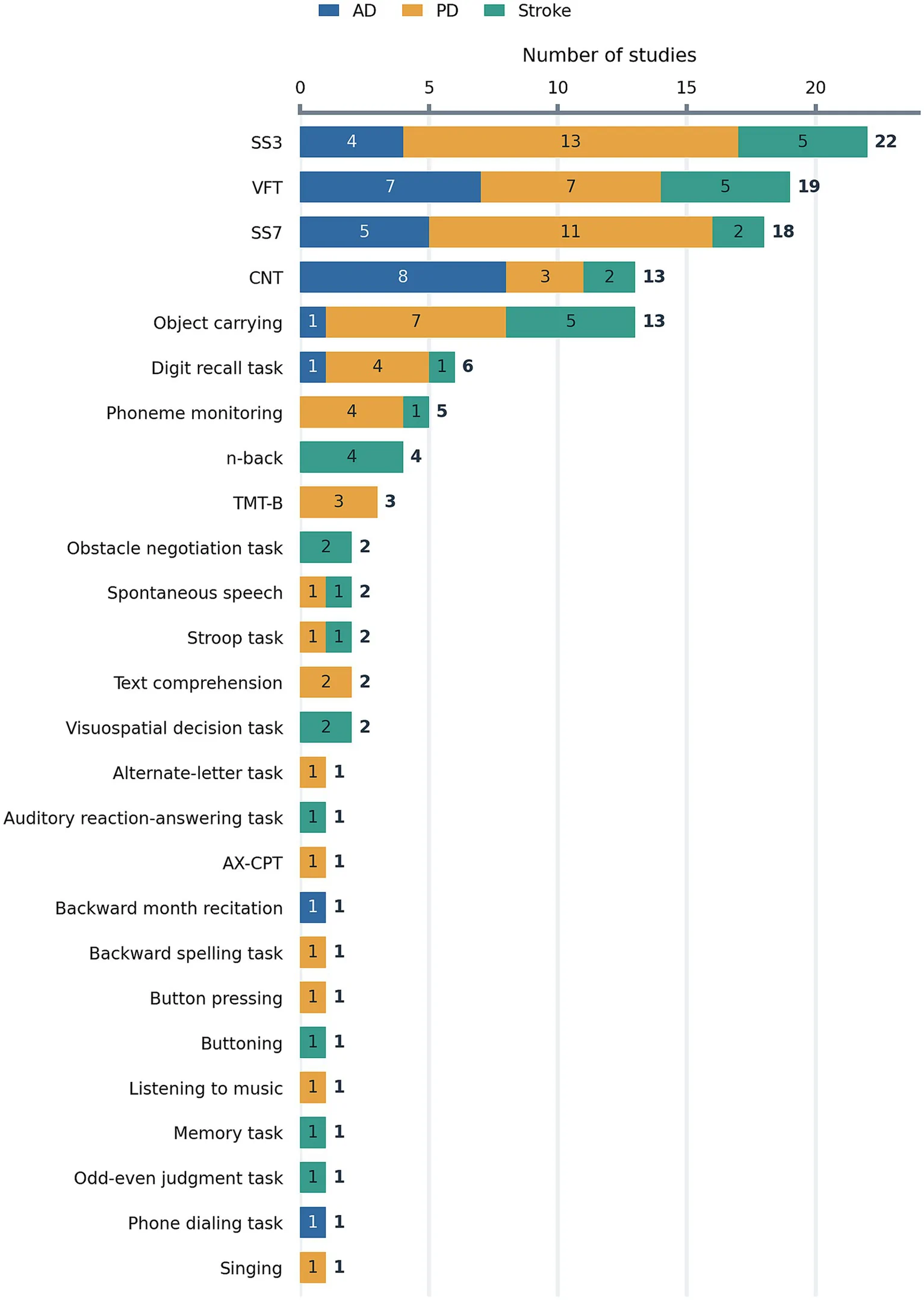
Distribution of DT paradigms across disease groups in the 84 included studies. The horizontal axis represents the number of included studies, and the vertical axis represents the 26 identified DT paradigms. Different colors represent studies involving AD, PD, and stroke. Each bar segment represents the number of unique studies in which the corresponding paradigm was used for a given disease group. Because an individual study could use more than one DT paradigm, the sum of the paradigm-specific study counts exceeds the total number of included studies. DT, dual task; AD, Alzheimer’s disease; PD, Parkinson’s disease; SS3, serial subtraction by 3s; SS7, serial subtraction by 7s; CNT, counting task; VFT, verbal fluency task; AX-CPT, AX Continuous Performance Test; TMT-B, Oral Trail Making Test Part B.

By paradigm, SS3 was the most frequently used, appearing in 22 studies, of which four, 13, and five involved patients with AD, patients with PD, and patients with stroke, respectively. This was followed by VFT (19 studies) and SS7 (18 studies). CNT and the object-carrying task were each used in 13 studies. CNT was used primarily in AD studies (eight studies), whereas the object-carrying task was more frequently used in PD (seven studies) and stroke studies (five studies).

By disease, the most frequently used paradigms in PD studies were SS3 and SS7, appearing in 13 and 11 studies, respectively. In AD studies, CNT and VFT were the most frequently used paradigms, appearing in eight and seven studies, respectively. In stroke studies, VFT, SS3, and the object-carrying task were each used in five studies. In addition to these commonly used paradigms, the digit recall task was used in six studies, the phoneme monitoring task in five studies, the n-back task in four studies, and the oral Trail Making Test Part B in three studies. Each of the remaining paradigms was examined in only 1–2 studies, indicating that the use of DT paradigms was unevenly distributed across diseases. Because an individual study could employ more than one DT paradigm, the sum of the numbers of studies corresponding to each paradigm exceeded the total number of included studies.

### 3.5 Differences in gait performance across dual-task paradigms in patients with central nervous system diseases

This section selected 52 studies from the 84 included studies for analysis according to the study selection criteria described in Section 2.4. Among them, 13 were AD-related studies, 28 were PD-related studies, and 11 were stroke-related studies. In addition, 21 studies reported HC data while also reporting data from disease groups. Based on the data extracted from these 52 studies, gait speed, stride length, and cadence were the three gait parameters included in the descriptive synthesis, and DTC was used to quantify the degree of parameter changes in DT relative to ST. The differences in gait characteristics of different disease groups and HC under four commonly used DT paradigms, namely SS3, SS7, CNT, and VFT, were then summarized and analyzed. The specific descriptive statistical results are shown in Table 2 and visualized in Figure 3.

**Figure 3:**
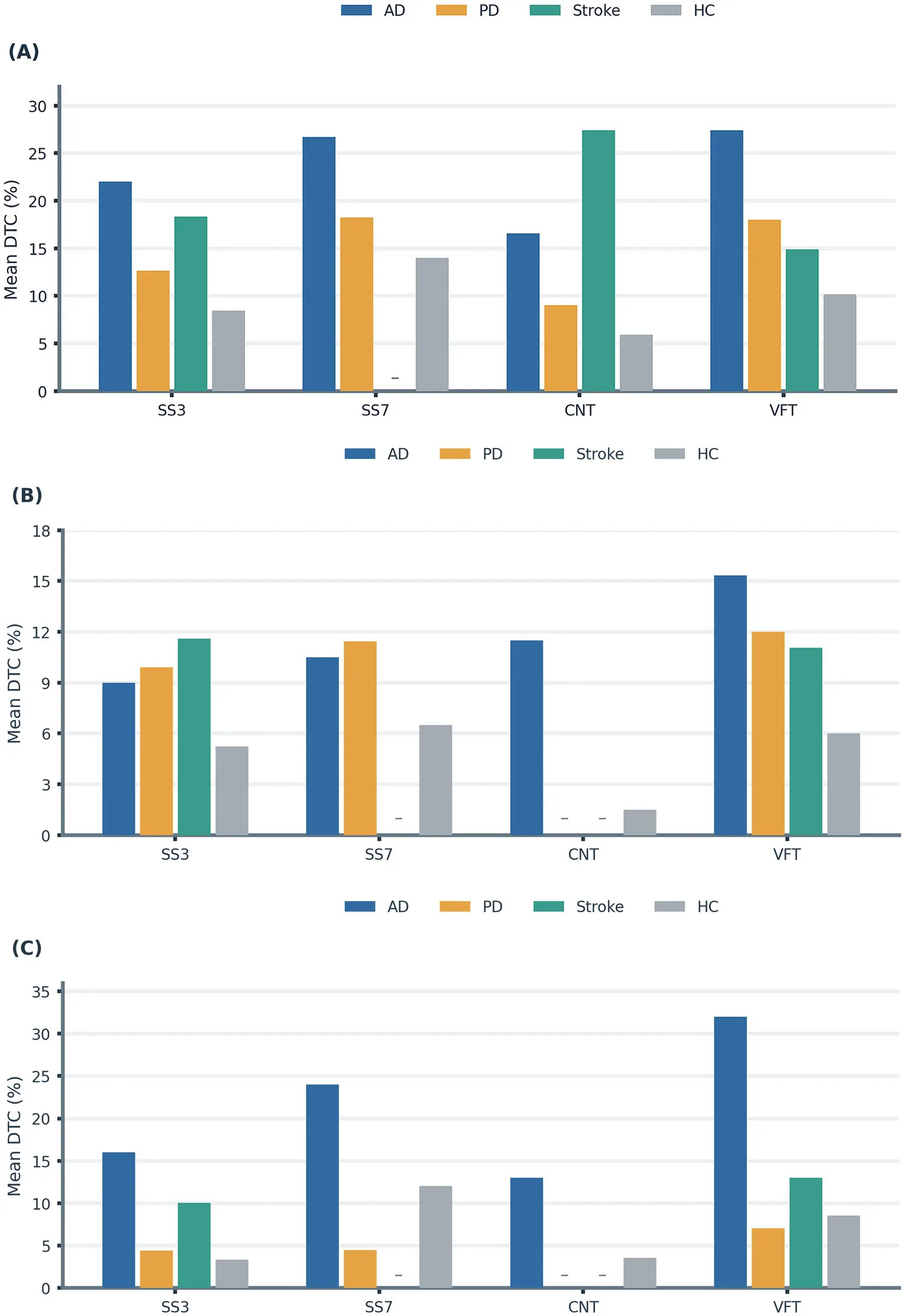
Descriptive mean DTC values for spatiotemporal gait parameters across DT paradigms. The three subplots show the distribution of mean DTC values for gait speed, stride length, and cadence, respectively. The *x*-axis represents the four DT paradigms: SS3, SS7, CNT, and VFT. Different colors represent different participant groups, including AD, PD, stroke, and HC. The values in the figure were plotted based on the mean values in Table 2 and are intended only for descriptive visualization; they do not indicate statistically significant differences. DT, dual task; DTC, dual-task cost; SS3, serial subtraction by 3s; SS7, serial subtraction by 7s; CNT, counting task; VFT, verbal fluency task; AD, Alzheimer’s disease; PD, Parkinson’s disease; HC, healthy controls. **(A)** Gait speed DTC. **(B)** Stride length DTC. **(C)** Cadence DTC.

In addition, to avoid misunderstanding the descriptive mean DTC values in Table 2 as statistically significant results, this study further summarized the reporting of statistical significance for gait parameter differences under ST–DT conditions in these 52 studies. The results showed that, for gait speed, 28 studies reported significant ST–DT differences, and three studies reported both significant and non-significant results across different participant groups or experimental conditions. For stride length, 14 studies reported significant ST–DT differences, and only one study reported non-significant ST–DT differences. For cadence, 10 studies reported significant ST–DT differences, and three studies reported both significant and non-significant results across different participant groups or experimental conditions. The detailed results are shown in Supplementary Table S2.

Table 2 presents changes in gait characteristics across participant groups under different DT conditions. To further describe the patterns across disease groups and DT paradigms, the results were organized into three aspects: changes in gait characteristics within the same participant group under different DT conditions, changes in gait characteristics across participant groups under the same DT, and overall patterns across different participant groups and DT conditions.

#### 3.5.1 Gait changes across dual-task paradigms within the same participant group

Among combinations with available data, descriptive mean DTC values varied across DT paradigms within the same participant group. As shown in Table 2:

(1) For patients with AD, gait speed DTC was highest under VFT-DT, at 27.42%, followed by SS7-DT (26.68%), SS3-DT (22.00%), and CNT-DT (16.56%). Stride length DTC was highest under VFT-DT, at 15.33%, followed by CNT-DT (11.50%), SS7-DT (10.50%), and SS3-DT (9.00%). Cadence DTC was highest under VFT-DT, at 32.00%, followed by SS7-DT (24.00%), SS3-DT (16.00%), and CNT-DT (13.00%). Gait speed DTC and stride length DTC under SS3-DT were each derived from one study. The cadence DTC values under SS7-DT, CNT-DT, and VFT-DT were also each derived from one study.
(2) For patients with PD, based on descriptive mean values, gait speed DTC was highest under SS7-DT, at 18.24%, which was close to that under VFT-DT (18.00%), followed by SS3-DT (12.64%) and CNT-DT (9.00%). Stride length DTC was highest under VFT-DT, at 12.00%, followed by SS7-DT (11.45%) and SS3-DT (9.90%). Cadence DTC was highest under VFT-DT, at 7.00%, followed by SS7-DT (4.46%) and SS3-DT (4.36%). No stride length or cadence DTC data were available under CNT-DT.
(3) For patients with stroke, based on descriptive mean values, gait speed DTC was highest under CNT-DT, at 27.42%, followed by SS3-DT (18.35%) and VFT-DT (14.89%). Stride length DTC under SS3-DT was 11.61%, which was close to that under VFT-DT (11.05%). Cadence DTC under VFT-DT was 13.00%, which was higher than that under SS3-DT (10.01%). Cadence DTC under VFT-DT was derived from one study. Available CNT-DT data were limited to gait speed DTC, and no gait speed, stride length, or cadence DTC data were available under SS7-DT.
(4) For HC, gait speed DTC was highest under SS7-DT, at 13.98%, followed by VFT-DT (10.14%), SS3-DT (8.45%), and CNT-DT (5.92%). Stride length DTC was highest under SS7-DT, at 6.50%, followed by VFT-DT (6.01%), SS3-DT (5.22%), and CNT-DT (1.50%). Cadence DTC was highest under SS7-DT, at 12.00%, followed by VFT-DT (8.55%), CNT-DT (3.50%), and SS3-DT (3.30%). Cadence DTC under SS7-DT was derived from one study.

#### 3.5.2 Gait changes across different participant groups under the same dual-task paradigm

Descriptive mean DTC values varied across participant groups under the same DT paradigm. It should be noted that some gait DTC data were derived from only one study or were missing (as indicated by “–” in Table 2), which may affect the reliability of some comparisons. The specific situations have been described in Section 3.5.1 and will not be repeated in this section. As shown in Table 2:

(1) Under SS3-DT, gait speed DTC was highest in patients with AD, at 22.00%, followed by patients with stroke (18.35%), patients with PD (12.64%), and HC (8.45%). Stride length DTC was highest in patients with stroke, at 11.61%, followed by patients with PD (9.90%), patients with AD (9.00%), and HC (5.22%). Cadence DTC was highest in patients with AD, at 16.00%, followed by patients with stroke (10.01%), patients with PD (4.36%), and HC (3.30%).
(2) Under SS7-DT, gait speed DTC was highest in patients with AD, at 26.68%, followed by patients with PD (18.24%) and HC (13.98%). Stride length DTC was highest in patients with PD, at 11.45%, followed by patients with AD (10.50%) and HC (6.50%). Cadence DTC was highest in patients with AD, at 24.00%, followed by HC (12.00%) and patients with PD (4.46%).
(3) Under CNT-DT, gait speed DTC was highest in patients with stroke, at 27.42%, followed by patients with AD (16.56%), patients with PD (9.00%), and HC (5.92%). Stride length DTC was 11.50% in patients with AD, which was higher than that in HC (1.50%). Cadence DTC was 13.00% in patients with AD, which was higher than that in HC (3.50%).
(4) Under VFT-DT, gait speed DTC was highest in patients with AD, at 27.42%, followed by patients with PD (18.00%), patients with stroke (14.89%), and HC (10.14%). Stride length DTC was highest in patients with AD, at 15.33%, followed by patients with PD (12.00%), patients with stroke (11.05%), and HC (6.01%). Cadence DTC was highest in patients with AD, at 32.00%, followed by patients with stroke (13.00%), HC (8.55%), and patients with PD (7.00%).

#### 3.5.3 Gait changes of different participant groups under different dual-task paradigms

The above sections separately summarized gait changes within the same participant group across different DT paradigms and gait changes across different participant groups under the same DT paradigm. In this section, based on the relatively more widely available data of gait speed DTC, gait speed DTC patterns across participant groups and DT paradigms were further summarized.

First, within each participant group, descriptive mean gait speed DTC varied across DT paradigms, and the pattern of variation differed among participant groups. Specifically, (1) qualitatively, for patients with AD, gait interference was more severe in VFT-DT. For patients with PD and HC, gait interference was more severe in SS7-DT. For patients with stroke, gait interference was more severe in CNT-DT. (2) quantitatively, for patients with AD, compared with HC, the mean gait speed DTC was higher by 10.64 percentage points in CNT-DT, higher by 13.55 percentage points in SS3-DT, higher by 12.70 percentage points in SS7-DT, and higher by 17.28 percentage points in VFT-DT. For patients with PD, compared with HC, the mean gait speed DTC was higher by 3.08 percentage points in CNT-DT, higher by 4.19 percentage points in SS3-DT, higher by 4.26 percentage points in SS7-DT, and higher by 7.86 percentage points in VFT-DT. For patients with stroke, compared with HC, the mean gait speed DTC was higher by 21.50 percentage points in CNT-DT, higher by 9.90 percentage points in SS3-DT, and higher by 4.75 percentage points in VFT-DT.

Second, within each DT paradigm, descriptive mean gait speed DTC varied across participant groups, and the pattern of variation differed among DT paradigms. Specifically, (1) qualitatively, in CNT-DT, gait interference was more severe in patients with stroke, while in SS3-DT, SS7-DT, and VFT-DT, gait interference was more severe in patients with AD. (2) quantitatively, in SS3-DT, compared with HC, the mean gait speed DTC was higher by 13.55 percentage points in patients with AD, higher by 4.19 percentage points in patients with PD, and higher by 9.90 percentage points in patients with stroke. In SS7-DT, compared with HC, the mean gait speed DTC was higher by 12.70 percentage points in patients with AD and higher by 4.26 percentage points in patients with PD. In CNT-DT, compared with HC, the mean gait speed DTC was higher by 10.64 percentage points in patients with AD, higher by 3.08 percentage points in patients with PD, and higher by 21.50 percentage points in patients with stroke. In VFT-DT, compared with HC, the mean gait speed DTC was higher by 17.28 percentage points in patients with AD, higher by 7.86 percentage points in patients with PD, and higher by 4.75 percentage points in patients with stroke.

Third, the relationship among gait speed, cadence, and stride length is gait speed = cadence × stride length. Taking patients with AD and HC, for whom the DTC data of gait speed, stride length, and cadence were relatively more widely available, as the target groups, the DTC change trends of gait speed and cadence were similar, while the DTC change trend of stride length was relatively flat. Therefore, it can indirectly indicate that the slowing effect of different DT on gait speed may mainly result from the decrease in cadence.

## 4 Discussion

Cognitive impairment in patients with CNS diseases is closely associated with gait characteristics (Plummer-D’Amato et al., 2008; Muir et al., 2012; Russo et al., 2023), and a large number of existing studies have explored gait characteristics of patients with different CNS diseases from multiple perspectives using various DT. However, the available evidence remains dispersed across different diseases and task contexts, and studies differ in the DT paradigms selected, gait parameters assessed, and DT implementation methods. These differences increase the difficulty of comparing and interpreting findings across studies. In response to the above problems, this study identified and classified the DT paradigms used in DT gait studies of AD, PD, and stroke; described the application and distribution of these paradigms across studies of different diseases; further examined changes in gait characteristics under different DT paradigms and considered the possible relationships between the cognitive demands of DT paradigms and disease-related cognitive impairment when interpreting these patterns.

Whether in HC or in patients with CNS diseases, when performing DT, an increase in gait speed DTC is observed, that is, gait performance under DT is worse than that under ST. This phenomenon can be explained based on the bottleneck theory of the brain, that is, the brain’s central processing system can only process one task serially at a time, and performing multiple tasks simultaneously is essentially switching behavior between different tasks (Pashler, 1984, 1994; Navon and Miller, 2002). Therefore, in DT, because participants need to switch frequently between different tasks, gait speed DTC increases. Furthermore, because patients with CNS diseases have different degrees of brain impairment, the switching efficiency between tasks is lower than that of HC, which may be the reason why gait speed DTC in patients with CNS diseases is higher than that in HC. For example, Lin et al. conducted an SS3-DT in patients with AD and HC, and their gait speed DTC values were 11% and 7%, respectively (Lin et al., 2016); Li et al. conducted an SS7-DT in patients with AD and HC, and their gait speed DTC values were 22% and 11%, respectively (Li et al., 2023).

Under different DT paradigms, patterns of gait change differed between patients with CNS diseases and HC, as well as among patients with different CNS diseases. For example, VFT-DT, SS7-DT, SS3-DT, and CNT-DT, which are four commonly used DT paradigms in gait research, were associated with increases of varying magnitudes in gait speed DTC across participant groups, as shown in Table 2. The following discussion interprets this pattern from the perspectives of the cognitive demands of different DT paradigms and cognitive impairment in patients with different CNS diseases.

First, the cognitive tasks used in different DT paradigms place different cognitive demands on the brain (Tsang et al., 2022; Wang et al., 2023). VFT mainly relies on language function for lexical retrieval and memory function for information storage (Woollacott and Shumway-Cook, 2002; Beauchet et al., 2005; Galletly and Brauer, 2005; Al-Yahya et al., 2011; Muir et al., 2012; Baetens et al., 2013; Whiteside et al., 2016). Serial subtraction mainly relies on executive function for rule-based numerical calculation (Beauchet et al., 2005; Galletly and Brauer, 2005; Muir et al., 2012; Baetens et al., 2013). CNT mainly relies on attention to maintain continuous number output (Muir et al., 2012; Oh et al., 2021). According to previous studies, among the cognitive tasks involved in this study, SS7 and VFT imposed relatively higher cognitive loads, whereas SS3 and CNT imposed relatively lower cognitive loads (Woollacott and Shumway-Cook, 2002; Mirelman et al., 2014; Li et al., 2018). In this study, the descriptive mean gait speed DTC in HC was highest under SS7-DT, at 13.98%, followed by VFT-DT (10.14%), SS3-DT (8.45%), and CNT-DT (5.92%). Therefore, these descriptive findings suggest that gait speed DTC in HC may be influenced primarily by the cognitive load imposed by the concurrent cognitive task.

Second, gait speed DTC in patients with CNS diseases is not only influenced by the cognitive load of the cognitive task, but is also related to their cognitive impairment.

(1) Based on descriptive mean values, gait speed DTC in patients with AD was highest under VFT-DT, at 27.42%, followed by SS7-DT (26.68%), SS3-DT (22.00%), and CNT-DT (16.56%). Compared with HC, gait speed DTC of patients with AD was the highest under VFT-DT, and the gait speed DTC under each DT was higher than that in HC. This phenomenon can be explained from the perspective of cognitive impairment in patients with AD. The typical cognitive impairments in patients with AD are impaired memory function and language function, which may be associated with lower task-switching efficiency during VFT-DT than during other tasks, thus showing higher gait speed DTC (Nebes, 1989; Andrade-Guerrero et al., 2024; Chen et al., 2025). In addition, gait speed DTC of patients with AD under all DT was higher than that of HC, which may be due to the overall decline in central cognitive function in patients with AD (Stern, 2012; Tenchov et al., 2024).
(2) Based on descriptive mean values, gait speed DTC in patients with PD was highest under SS7-DT, at 18.24%, but was close to that under VFT-DT (18.00%), followed by SS3-DT (12.64%) and CNT-DT (9.00%). Patients with PD have overall impairment of central cognitive function (Kvist et al., 2024; Zhou et al., 2025), and their typical cognitive impairment is impaired motor control function (Cristini et al., 2023; Zhai et al., 2023). Compared with patients with AD, patients with PD have more severe impairment of motor control function. Therefore, the ranking of gait speed DTC of patients with PD under different DT was consistent with that of HC, but gait speed DTC under all DT was higher than that of HC. However, gait speed DTC of patients with PD under different DT was lower than that of patients with AD. This indicates that, under DT, the degree of impairment of central cognitive function in patients with PD is lower than that in patients with AD, which is sufficient to compensate to some extent for the more severe impairment of motor control function in patients with PD.
(3) Compared with AD and PD, cognitive impairment in patients with stroke exhibits greater heterogeneity, and the type and severity of cognitive impairment in patients with stroke are not only related to lesion volume and number, but also related to the specific anatomical location of lesions (Rost et al., 2022; Tsiakiri et al., 2024). Therefore, in different studies, even under the same DT paradigm, patients with stroke may exhibit different gait speed DTC due to differences in participant characteristics. For example, Chatterjee et al. conducted VFT-DT in patients with stroke, and the gait speed DTC was 7% (Chatterjee et al., 2018); Hawkins et al. also conducted VFT-DT in patients with stroke, and the gait speed DTC was 35.5% (Hawkins et al., 2018). In this study, based on descriptive mean values, gait speed DTC in patients with stroke was highest under CNT-DT, at 27.42%, followed by SS3-DT (18.35%) and VFT-DT (14.89%), and a larger gait speed DTC occurred in CNT-DT, which had the lowest cognitive load. The reasons for the above results are currently difficult to determine, but we speculate that there may be the following reasons: (1) the impact on gait performance due to differences in lesion characteristics of patients with stroke in different studies; (2) the cognitive load of SS3-DT and VFT-DT is too high for patients with stroke, resulting in abandonment of the cognitive task by patients; (3) differences in DT prioritization across studies, that is, some participants prioritize the gait task, while others prioritize the cognitive task.

Third, in addition to disease type and DT paradigm, the selection of gait parameters may also influence the presentation and interpretation of study findings because different gait parameters reflect distinct dimensions of gait control and may vary in their sensitivity to DT interference. For example, Oh et al. assessed the Functional Ambulation Performance score, stride length, gait speed, and double-support time in patients with AD. The results showed that, compared with the ST baseline model, adding Functional Ambulation Performance, stride length, or double-support time measured under VFT-DT to the model significantly improved model fit, whereas adding gait speed measured under the same condition did not significantly improve model fit (Oh, 2021). This finding suggests that if gait speed alone were used as the gait parameter, researchers might conclude that VFT-DT provides limited additional value over ST walking for identifying patients with AD. However, the inclusion of other gait parameters that are more sensitive to DT interference may yield different findings and interpretations. Similarly, even within the same study population, DT condition, and between-group comparison framework, gait parameters may differ in their sensitivity to between-group differences. Yang et al. assessed gait speed, cadence, walking time, stride length, the temporal symmetry index, and the spatial symmetry index in patients with stroke. Compared with HC, only the magnitude of the DT-related decline in stride length differed significantly between groups under the buttoning-task DT condition. Under the tray-carrying DT condition, however, the magnitudes of the DT-related declines in gait speed, stride length, and the temporal symmetry index all differed significantly between groups (Yang et al., 2007). These findings indicate that selecting only a single gait parameter may fail to capture DT-related changes in other dimensions of gait, thereby affecting judgments regarding DT effects and between-group differences. Therefore, gait parameter selection may influence whether an effect is detected, its estimated magnitude, and the interpretation of study conclusions, while further reducing direct comparability across studies.

Based on the above results and discussion, we provide the following considerations for future DT experiments in patients with CNS diseases.

(1) In experimental design, cognitive impairment in patients with CNS diseases and the cognitive demand characteristics of DT should be considered (Galletly and Brauer, 2005; Plummer-D’Amato et al., 2008). For example, patients with AD have severe impairment of language function (Chen et al., 2025), and VFT-DT may cause greater gait interference (Oh, 2021); Patients with PD have more severe impairment of motor control function (Galletly and Brauer, 2005), and an object-carrying DT, such as carrying a cup of water, may cause greater gait interference (Liu et al., 2022); for patients with stroke, cognitive impairment should first be determined according to lesion type (Rost et al., 2022), and DT paradigms that place demands on their impaired cognitive functions may cause greater gait interference (Plummer-D’Amato et al., 2008).
(2) During DT experiments, participants’ task-prioritization strategies should be considered. Specifically, researchers should determine whether participants prioritize the cognitive task or the walking task when performing a cognitive DT (Plotnik et al., 2011; Wild et al., 2013). If the prioritization issue is not considered in experimental design, participants may show different prioritization strategies during DT, thus affecting the reliability and comparability of experimental results (Kim et al., 2025). Consideration of this issue in previous studies has been relatively insufficient, which may also be one of the reasons for the large differences in results among similar previous studies.
(3) In cognitive DT, both the cognitive task and the walking task are present, but previous studies mainly focused on gait parameters in the walking task, and attention to cognitive task performance has been insufficient. Paying attention to both gait parameters and cognitive task performance not only helps to observe the allocation of brain resources to different tasks in a given participant during the experiment, but also allows comparison of differences in cognitive task completion among participants. Therefore, we suggest that future related studies, in addition to collecting gait parameters, should also examine cognitive task performance such as response time and accuracy (Galletly and Brauer, 2005).

Finally, this study also has certain limitations.

(1) The included studies differed in participant characteristics, disease stage, cognitive status, DT paradigm and implementation methods, task-prioritization instructions, gait assessment protocols and equipment, and the definitions and reporting methods of gait parameters. In addition, different gait parameters may vary in their sensitivity to DT conditions. These sources of clinical and methodological heterogeneity limited direct comparisons across diseases and DT paradigms and may also have contributed to inconsistencies in the study findings.
(2) The findings of the methodological appraisal should be considered when interpreting the results of this review. Although outcome measurement and statistical analysis were generally appropriate across the included studies, insufficient reporting of eligibility criteria, diagnostic standards, confounder control, follow-up, and randomization procedures reduced confidence in some between-study comparisons. Future dual-task gait studies should prespecify eligibility and diagnostic criteria, appropriately address relevant confounding factors, improve reporting transparency, and assess both gait performance and concurrent-task performance.
(3) The descriptive synthesis of DTC in this study was limited to four commonly used DT paradigms—CNT, SS3, SS7, and VFT—and three gait parameters—gait speed, cadence, and stride length—for which extractable data were more frequently available. Other paradigms, such as object-carrying tasks, were not included in this analysis, which may limit the applicability of the findings. In addition, unweighted mean DTC values were used without accounting for study sample sizes or the precision of the estimates, and pooled effect sizes and their uncertainty were not calculated. Therefore, these results only reflect potential patterns of variation in the existing literature and cannot be used to test for statistical differences between diseases or paradigms or to determine the relative advantages of different paradigms. The relatively high gait speed DTC observed among patients with AD under certain task conditions may also have been influenced by task type, the uneven number of available studies, and differences in study characteristics.
(4) The selection of a DT paradigm depends not only on the type of CNS disease but also on the specific research objectives, the experimental variables to be manipulated, and the gait or cognitive parameters to be assessed. This review only identified and categorized the DT paradigms used in DT gait studies of AD, PD, and stroke and further explored changes in gait characteristics under different DT conditions; it did not systematically evaluate the suitability of each paradigm for different research contexts. Therefore, the findings may help future studies identify factors that should be considered in experimental design but cannot be used to establish specific criteria for paradigm selection.
(5) In accordance with the study objectives and predefined scope, this study included only three CNS diseases: AD, PD, and stroke. However, multiple sclerosis, Huntington’s disease, and amyotrophic lateral sclerosis are also important CNS diseases. Patients with these diseases may also experience motor, sensory, and cognitive impairments, as well as deficits in balance control, all of which may affect DT walking performance. Therefore, when sufficient evidence becomes available, future studies could further systematically synthesize DT gait research involving other CNS diseases to broaden the understanding of DT gait mechanisms across different CNS diseases.

## 5 Conclusion

This scoping review showed that DT gait studies in AD, PD, and stroke have employed a wide range of DT paradigms. However, their use was concentrated in a small number of commonly used paradigms and was unevenly distributed across studies of the three diseases. The exploratory descriptive analysis of major spatiotemporal gait parameters under several commonly used DT paradigms indicated that the patterns of gait changes reported in existing studies may differ according to disease type, DT paradigm, and the gait parameter assessed. These findings suggest that the results of DT gait studies should be interpreted in the context of the specific disease population, DT paradigm, and gait parameter.

In future DT gait studies involving patients with CNS diseases, experimental design should take into account the specific research objectives, disease type, the cognitive or motor demands of the DT paradigm, and the variables that researchers intend to manipulate or assess. Researchers should also select gait parameters aligned with their research objectives, clearly report DT implementation methods and task-prioritization instructions, and assess cognitive-task performance concurrently whenever feasible. As the evidence base for gait research in CNS diseases continues to develop, future research could further examine the relationships among disease type, DT paradigm, and gait parameter and evaluate the suitability of specific paradigms for particular research contexts.

## Data Availability

The raw data supporting the conclusions of this article will be made available by the authors, without undue reservation.
